# Tricyclodecan-9-yl-Xanthogenate (D609): Mechanism of Action and Pharmacological Applications

**DOI:** 10.3390/ijms23063305

**Published:** 2022-03-18

**Authors:** Aashiq Hussain Bhat, Khalid Bashir Dar, Andleeb Khan, Saeed Alshahrani, Sultan M. Alshehri, Mohammed M. Ghoneim, Prawez Alam, Faiyaz Shakeel

**Affiliations:** 1 Department of Clinical Biochemistry, University of Kashmir, Srinagar 190006, India; aashiqbht8@gmail.com (A.H.B.); khalidnigeeni@gmail.com (K.B.D.); 2 Department of Pharmacology and Toxicology, College of Pharmacy, Jazan University, Jazan 45142, Saudi Arabia; saalshahrani@jazanu.edu.sa; 3 Department of Pharmaceutics, College of Pharmacy, King Saud University, Riyadh 11451, Saudi Arabia; salshehri1@ksu.edu.sa (S.M.A.); faiyazs@fastmail.fm (F.S.); 4 Department of Pharmacy Practice, College of Pharmacy, AlMaarefa University, Ad Diriyah 13713, Saudi Arabia; mghoneim@mcst.edu.sa; 5 Department of Pharmacognosy, College of Pharmacy, Prince Sattam Bin Abdulaziz University, Al-Kharj 11942, Saudi Arabia; prawez_pharma@yahoo.com

**Keywords:** tricyclodecan-9-yl xanthogenate (D609), cellular proliferation, ceramide, pharmacological properties

## Abstract

Tricyclodecan-9-yl xanthogenate (D609) is a synthetic tricyclic compound possessing a xanthate group. This xanthogenate compound is known for its diverse pharmacological properties. Over the last three decades, many studies have reported the biological activities of D609, including antioxidant, antiapoptotic, anticholinergic, anti-tumor, anti-inflammatory, anti-viral, anti-proliferative, and neuroprotective activities. Its mechanism of action is extensively attributed to its ability to cause the competitive inhibition of phosphatidylcholine (PC)-specific phospholipase C (PC-PLC) and sphingomyelin synthase (SMS). The inhibition of PCPLC or SMS affects secondary messengers with a lipidic nature, i.e., 1,2-diacylglycerol (DAG) and ceramide. Various in vitro/in vivo studies suggest that PCPLC and SMS inhibition regulate the cell cycle, block cellular proliferation, and induce differentiation. D609 acts as a pro-inflammatory cytokine antagonist and diminishes Aβ-stimulated toxicity. PCPLC enzymatic activity essentially requires Zn^2+^, and D609 might act as a potential chelator of Zn^2+^, thereby blocking PCPLC enzymatic activity. D609 also demonstrates promising results in reducing atherosclerotic plaque formation, post-stroke cerebral infarction, and cancer progression. The present compilation provides a comprehensive mechanistic insight into D609, including its chemistry, mechanism of action, and regulation of various pharmacological activities.

## 1. Introduction

**Figure 1:**
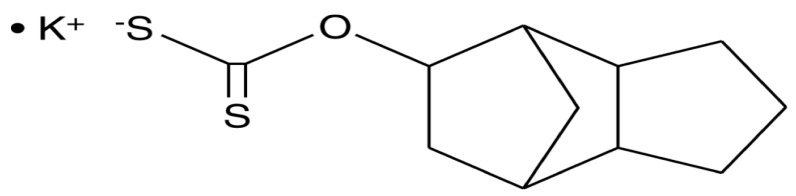
Structure of tricyclodecan-9-yl xanthogenate (D609).

Tricyclodecan-9-yl xanthogenate (D609) is a synthetic tricyclic compound featuring a xanthate group, known as a phosphate group analog (Figure 1). The first antiviral biological experiments did not commence until 1984, over fifty years after the initial synthesis was published [1,2]. The discovery of the various biological mechanisms that followed led to the detection of many other biological mechanisms, including anti-tumoral, antiviral, anti-apoptotic, and anti-inflammatory effects [3,4,5]. The D609 suppressed both acidic sphingomyelinase and phosphatidylcholine-specific phospholipase C (PC-PLC) activity due to its unique competitive inhibitory action on both these enzymes [6,7].

Recently, D609 was observed to be the single recorded sphingomyelin synthase (SMS) inhibitor that correlates with the clearance of amyloid-β peptide as well as metabolic syndrome [8]. Over the last 30 years, more than 700 reports on the biological activity of D609 have been published, predominantly for its lipid biology [9]. Essentially, D609 is an extremely sturdy regulator of lipid biology and lipid chemical biology [10]. There are eight stereoisomers and their enantiomers paired by three asymmetric centers in D609 [11,12]. The biological activities of D609 are typically stimulated by phosphatidylcholine (PC)-specific inhibition of phospholipase C (PC-PLC) [13,14]. The suppression of PC-PLC and/or SMS by D609 can be averted by lipid second messengers; ceramide and/or 1,2-diacylglycerol (DAG) [15,16]. According to reports, the inhibition of either SMS or PC-PLC regulated the cell cycle in various in vitro and in vivo experiments and suppressed proliferation while promoting differentiation. Xanthogenate compounds, including D609, possess potent antioxidant properties, and D609 also diminishes the Aß-stimulated toxicity [17,18,19]. Zn^2+^ is necessary for the enzymatic activity of PC-PLC, while chelation with Zn^2+^ leads to the suppression of D609 [20]. D609 also has a role in suppressing acidic sphingomyelinase [21,22] or down-regulating hypoxia-inducible factor-1a, even though these are downstream actions imperative to the PC-PLC suppression [23]. PC-PLC mammals′ characterization is restricted to blocking enzymatic activity (commonly calculated with bacterial PC-PLC as a standard, called Amplex red assay) [6]. Until now, no cloning of PC-PLC mammals has occurred, and no systemic and temporal specifics are yet available. D609 exhibited promising results for decreasing atherosclerotic plaque formation (PC-PLC inhibition), as well as stroke infarction [24,25]. As a pro-inflammatory cytokine receptor, PC-PLC is responsible for the action of D609. Following oxidation, D609 possesses a free thiol group that releases a disulfide that mimics glutathione. The resulting molecule, which regenerates D609, is a glutathione reductase substratum [18]. D609 defends the brain and cultures of neurons from potential triggers of AD (Alzheimer′s disease) and oxidative stress caused by Aβ cytotoxicity, according to recent reports [18,26]. Mitochondria are vital organelles containing pro and anti-apoptotic protein factors. Under in vitro conditions, the administration of D609 is believed to mediate neuroprotection against apoptosis and free-radical-induced mitochondrial damage [18]. In vitro oxidants, Fe^2+^/H_2_O_2_ (hydroxyl free radicals), and AD relevant peptides 1–42 A (1–42) and 2,2-azobis (2-amidinopropane) dihydrochloride were used to recover brain mitochondria from gerbils 1 h after the intraperitoneal (*i.p.*) administration of D609 (AAPH, alkoxyl, and peroxyl free radicals).

Protein carbonyl, protein-bound hydroxynonenal (HNE) (a lipid peroxidation product), 3-nitrotyrosine (3-NT), and cytochrome-c release were all significantly lower in oxidant-treated brain mitochondria isolated from saline-injected gerbils [18]. Moreover, D609 therapy successfully maintained the (reduced glutathione/oxidized glutathione) GSH/GSSG ratio of oxidant-treated mitochondria. D609-treated glutathione reductase (GR), glutathione peroxidase (GPx), and glutathione S-transferase (GST) gerbils improved brain growth, which reinforced the concept that D609 works as GSH. The anti-apoptotic properties of this molecule are studied as possible therapeutics for oxidative stress-induced neurodegenerative diseases, such as AD [27].

In this review, a comprehensive analysis of the mechanism of D609 and its pharmacological action in different serious ailments and its role is discussed in detail. A large proportion of the study is devoted to the mechanism of D609 as an antiviral, antioxidant, anti-inflammatory, anti-tumoral, antiapoptotic, anticholinergic activity which are comprehensively documented.

## 2. Chemistry of D609

D609 comprises a total of eight stereoisomers in addition to their enantiomers, which are allocated among three asymmetric centers in D609 [2,28]. The initial reports of D609 date back to the year 2002 [29], and, currently, the drug is also available from several commercial sources. Until now, no comprehensive study has been carried out to examine the impact on the biological behavior of D609s. In fact, due to the enormous difficulties of assigning proper stereochemistry to modern chemical analytics, no chiral or relative stereochemistry information is generally present in commercially available D609s. Vibrational circular dichroism (VCD) spectroscopy with ab initio theoretical computations has been developed during the last decade to determine the underlying structure of D609. Through the cellular vibrational transition, VCD estimates the differential absorption of circularly polarized IR radiation on the chiral molecule [11].

## 3. Mechanism of Action of D609

### 3.1. PC Specific-PC-PLC

**Figure 2:**
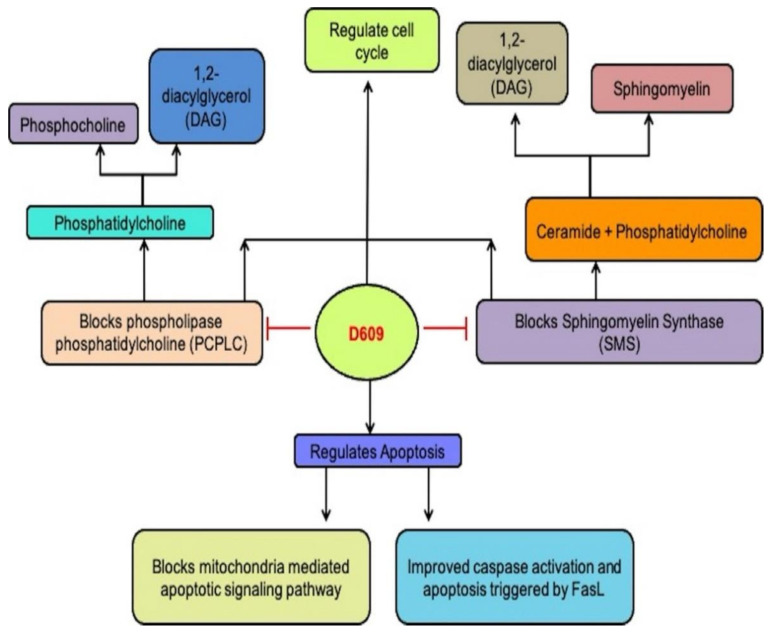
D609 blocks PCPLC and SMS and regulates cell cycle and apoptosis.

PC-PLC (66 kDa) hydrolyzes phosphocholine and 1,2-diacylglycerol PCs to produce DAG. Although scientists have purified bacterial PC-PLC, mammalian PC-PLC is yet to be cloned. Its sequence is still undetermined, thereby restricting the identification of D609 actions by a mammalian enzyme [30]. Rabbit polyclonal antibodies demonstrated cross-reactivity with mammalian PC-PLC to *Bacillus cereus* PC-PLCI in the occurrence or lack of an essential fibroblast growth factor (ßFGF). The suppression of PC-PLC by D609 blocked replication and permitted different cell systems to be distinguished (Figure 2). PC-PLC cells also have a role in vascular endothelial apoptosis and senesis. In a cell-cycle-dependent manner, the PC-PLC expression varied inversely with cell division cycle protein 20 (CDC20) homolog and CDC20 overexpression stimulated ubiquitin-proteasome pathway-mediated PC-PLC degradation [31]. A highly glycosylated transmembrane protein, cluster of differentiation-16 (CD16), also mediated PC-PLC expression in (natural killer) NK cells. D609 treatment caused a dramatic reduction in the expression of PC-PLC and CD16 receptor [32]. Acts of D609 due to PC-PLC inhibition include the inhibited post-stroke production of hypoxia-inducible factor 1-alpha (HIF-1 α), the protection of tumor necrosis factor (TNF-α) or lethal shock mediated by LPS in mice, diminished expression of cytokines in macrophages induced by lipopolysaccharide (LPS) and safeguarding immature neurons (non-expressed glutamate receptors) against oxidative toxic glutamate [33]. According to reports, inhibiting PC-PLC with D609 promotes phospholipase D (PLD) action in UMR-106 osteoblastic cells, which could be due to counterbalancing effects of D609 or direct enhancement of PLD [34].

Derivatives of xanthate fit into the active core of PC-PLC with a lipophilic chain, and the dithiocarbonate group possibly functions as a phosphate replacement and binds to the active site of Zn^2+^ ions [3,14,35,36]. Generally, Amplex Red assay is employed to monitor the operation of PC-PLC. This assay works on the theory that PC-PLC hydrolyzes DAG-PC and phosphocholine-PC. The enzyme alkaline phosphatase converts phosphocholine to choline. Choline oxidase is converted to betaine, which produces H_2_O_2_. The Amplex Red reagent is stoichiometrically oxidized by hydrogen peroxide in the presence of horseradish peroxidase (HRPO) to produce fluorescent resorufin, which can be measured spectrophotometrically or fluorometrically. [37,38].

### 3.2. Sphingolipid Metabolism

In de novo ceramide biosynthesis, serine palmitoyltransferase (SPT) catalyzes the first and rate-limiting step. In the form of SM and DAG, the phosphocholine group from PC is converted to ceramide in the PC-ceramide membrane framework. Golgi apparatus has SMS1, while plasma membrane has SMS2. These two types of SMS are inhibited by D609 [6,39,40,41,42]. D609′s effects on PC-PLC inhibition appear to include SMS inhibition, according to studies [43]. De novo ceramide production was similarly boosted by D609, which might be explained by SPT stimulation [44]. In one rat stroke model, D609′s neuroprotection was due to the inhibition of SMS, which induced the accumulation of ceramide and influenced cell-cycle events. Lipid rafts/microdomains and the transport of fatty acids to the cell are associated with scavenger CD36/fatty acid translocase. The translocation and function of CD36 can be impacted by the ceramide [6,45]. Levels of ceramide may be critical for its signaling and may induce retinoblastoma dephosphorylation, triggering the arrest of the cell cycle (Rb) [46,47,48]. D609 suppressed ßFGF-stimulated astrocyte spread, probably due to SMS inhibition and an elevation in levels of ceramide [49]. By inhibiting SMS and upregulating the cyclin-dependent kinase (Cdk) inhibitors p27 and p21, D609 can cause the cell-cycle arrest and increase ceramide levels [50,51,52]. Ceramide may stimulate p27 and p21 expression by activating c-myc regulating protein phosphatase 2A (PP2A), which suppresses p21 and p27 expression [53]. Since the expression of cellular myelocytomatosis oncogene (c-myc) is not blocked by D609, these downstream effects are likely to increase ceramide levels.

## 4. Pharmacological Properties of D609

### 4.1. Antioxidant

D609 is an effective antioxidant in numerous studies. As with Alzheimer’s disease (AD), many neurological disorders are linked to oxidative stress. Intracellular neurofibrillary tangles (NFTs), extracellular amyloid protein deposits (primarily consisting of hyperphosphorylated tau protein), mitochondrial malfunction, synapse degradation, and apoptosis or cell death are all symptoms of AD [18,54]. The reduced energy metabolism in AD could be due to the oxidative failure of some essential metabolic or mitochondrial enzymes, which would lead to increased ROS [55]. Mitochondria is well-known as a key cellular energy-producing organelle. The production of ROS, in turn, leads to neuronal oxidative damage, which is dependent on these organelles [56,57]. In mitochondria, specific anti-apoptotic signals and pro-apoptotic defenses unite. Protein factors such as the second mitochondria-derived activator of caspase (SMAC) protein, and direct inhibitor of apoptosis-binding protein with low pi (DIABLO), cytochrome-C, apoptosis-inducing factor (AIF), and apoptotic protease activating factor 1 (Apaf1) produced during mitochondrial oxidative stress trigger caspase-independent and caspase-dependent pathways, leading to programmed cell death [58]. Mitochondria regulate intracellular Ca^2+^ homeostasis, generate ATP, and produce endogenous ROS. A higher concentration of mitochondrial calcium leads to superoxide formation and pro-apoptotic mitochondrial protein release, culminating in cell death [59].

DNA, RNA, protein, and lipid peroxidation, as well as neuronal malfunction or death, all occur in the AD brains [60]. Current research suggests that lipid peroxidation and protein oxidation in brains with mild cognitive impairment indicate that oxidative stress occurs early in the AD pathogenesis [61]. Understanding oxidative stress and its associated diseases, necessitates a comprehensive understanding of mitochondrial ROS activity and its effect on neuronal processes. The transport of mitochondrial electrons may be a source of ROS [62]. ROS such as hydroxyl radical (OH), hydrogen peroxide (H_2_O_2_), peroxynitrite (ONOO^−^), and superoxide anion (O^2−^) have long been known to cause neurodegeneration [63]. The mitochondrial membrane potential promotes the release of cytochrome-C into the cytoplasm and enhances the activity of caspase-3, signifying that mitochondrial mtDNA-derived malfunction contributes to Alzheimer′s disease [64]. Many studies confirm D609 to be helpful in a variety of central nervous system (CNS) and other neurological disorders [18].

D609 exhibits antioxidant and glutathione mimic properties due to its thiol content. Reduced glutathione (GSH) has been studied for its mimetic/antioxidant activity in the Aß toxicity trials [16]. D609′s xanthate group can quickly oxidize into a disulfide, which is a glutathione reductase substrate for the D609 regeneration [26]. In a variety of neurological disorders and CNS traumas, ROS and lipid peroxidation/oxidation have been found to play a crucial role in tissue pathophysiology. D609′s antioxidant/glutathione mimetic properties reduced ROS and oxidized PC (OxPC) growth, thereby benefiting these CNS pathologies [18]; D609 is a compound with anti-inflammatory and antiviral properties [4]. The majority of these occurrences are related to D609′s inhibition of PC-PLC [65]. This inhibition prevents secondary messenger diacylglycerol (DAG) synthesis, which in turn inhibits PKC and acidic sphingomyelinase (aSMase) [66]. Although it is unclear which ROS species D609 can effectively scavenge, this xanthate can scavenge hydroxyl radicals. Since xanthates have the good reductive ability, they are likely to react with other ROS [18]. As with pyrrolidine dithiocarbamate, a well-known antioxidant; D609 also inhibits dihydrorhodamine Fenton-reaction-induced oxidation in a concentration-dependent manner. The free thiol moiety in xanthate anions and protonated xanthic acid makes them highly reductive [6,17]. D609 also prevents alpha-phenyl-tert-butylnitron free-radical spin adducts and synaptosomal membrane lipid peroxidation, when Fenton reactions are used [67]. D609 has been shown to protect intracellular GSH, a key intracellular defense molecule in neurons, from oxidative stress and radiation [17,68]. D609, mimetic glutathione, has recently been shown to protect primary neuronal cultures from amyloid-peptide (1–42) induced oxidative stress and neurotoxicity in vitro and in vivo synaptosomes [69,70].

### 4.2. Anti-Viral

D609 was created with the intention of turning it into an antiviral agent. D609 and other Xanthates were first described as broad-spectrum antiviral compounds [1]. D609 exists as an enantiomeric pair of four diastereomers, with unknown variable mixtures of these eight isomers in industrial preparations. In antiviral and PC-PLC inhibition assays, isomers have varying biochemical efficacy [65]. Lumavita (Basel, Switzerland) is working on an antiviral drug called Letermovir-601 (LMV-601), which is a pure enantiomeric isomer of D609. Over time, LMV-601, a PC-PLC inhibitor, decreased the high-risk Human Papillomavirus (HPV) expression in types 16, 18, and 31, and exacerbated defects of pre-cancer cervical cell [6]. Tricyclodecan-9-yl-xanthogenate, an antiviral xanthate compound, can inhibit RNA and DNA viruses in vitro [71]. Infectivity assays and Western blot analysis have shown that it can prevent infectious HIV from spreading to chronically infected lymphoma cell (KE37-III) tissue-culture medium [72]. HIV-specific proteins, on the other hand, have accumulated intracellularly. At D609 doses that allowed mitotic cell divisions, de novo HIV replication commencement after infection with permissive KE37-1 cells was entirely stopped. Furthermore, the loss of HIV replicative intermediate DNA demonstrated the xanthate compound′s selective anti-viral efficacy. Within these cells, the cellular gene expression of c-myc remains unchanged [72].

**Figure 3:**
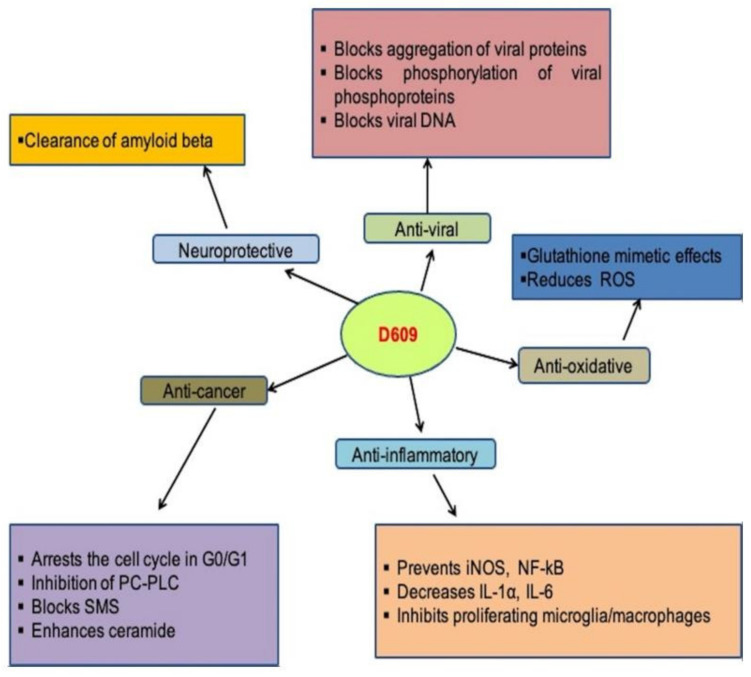
Role of D609 in human diseases and mechanisms involved.

The human respiratory syncytial virus (RSV) is a cytoplasmic, enveloped virus with negative RNA polarity. In newborns and young children, paramyxovirus is among the common causes of respiratory tract infections [73]. In human epithelial cells, the antiviral compound D609 prevents the development of the respiratory syncytial (RS) virus (Hep 2) [74]. Viral protein aggregation, the viral phosphorylation of phosphoprotein, infectious particles levels, and extracellular antigens all decreased after treatment with D609. When the polarity was positive or negative, the viral proteins’ relative accumulation was also unbalanced, but there were no variations in viral RNA volume. Furthermore, nucleocapsid formation was not hampered [75]. HSV-1 (Herpes simplex virus-1) replication was prevented by D609 without causing cytotoxicity. It inhibited HSV-1 encoded protein kinase and decreased virus-infected cell polypeptide phosphorylation (US3 PK). At concentrations greater than 3.8 µM, virus production decreased by D609, with absolute inhibition at 75.2 µM at or below 1 PFU/cell MOI. D609 can be given up to 7 h after infection and still prevent virus replication. These findings indicate that D609′s antiviral activity is mediated by the protein kinase inhibition and phosphorylation of protein, which influences late HSV replication. As a result of the above research, it is clear that D609 prevents the development of many virus-related diseases [76]. The role of D609 in various diseases and its mechanisms are summarized in Figure 3.

### 4.3. Anti-Inflammatory

D609 has also demonstrated significant potential anti-inflammatory activities [77]. Immunization of uveal melanin protein causes experimental melanin–protein induced uveitis (EMIU), which is a form of chronic autoimmune uveitis. The induction of inductive nitric oxide synthase (iNOS) is prevented by D609, a basic inhibitor of phosphatidylcholine-specific phospholipase C [78,79]. Thirty-five (Lewis) rats with EMIU were given either PBS or D609 in two separate experiments. D609 had a significant inhibitory effect on EMIU during this study [80]. D609-treated eyes had lower peroxide and nitrite levels, higher SOD levels, and lower iNOS mRNA expressions than PBS-treated eyes. The Fas and FasL levels in D609-injected animals′ eyes and lymph nodes increased significantly. D609-treated rats had increased DNA fragmentation in their lymph nodes. D609 inhibited EMIU by quenching NO and initiating programmed cell death, which had formerly been inhibited by NO, as well as displaying anti-inflammatory properties [79]. D609, a NO scavenger, was recently shown to be effective at preventing allergic encephalomyelitis in animals [81,82]. D609, in addition to inhibiting NO, can also inhibit other inflammatory mediators. By preventing the PC-PLC pathway, D609 inhibits the development of NOS activity. In response to inflammation, the PC-PLC pathway, which plays an important role, is activated [83,84]. D609 reduces IL-1α, IL-6, and NO release in endotoxin shock mice, according to Tschaikowsky et al. D609′s inhibition of NO synthesis has a much greater protective effect than D609′s inhibition of other inflammatory mediators. It has also been shown that D609, a PC-PLC and NOS inhibitor, inhibited NO expression in EMIU [85]. N9 and BV-2 microglia, RAW 264.7 macrophages, and DITNC1 astrocytes were all significantly inhibited by 100-μM D609 treatment without affecting cell viability [50]. D609 may perform an anti-inflammatory function by preventing the proliferation of macrophages/microglia, which are the principal sources of IL-1 and TNF and other pro-inflammatory cytokines. TNF-induced PAR formation is inhibited in D609-treated cultures, according to studies. D609 reduced microglial morphological change, NF-kB transcriptional activation, and PARP-1 activation [86].

### 4.4. Anti-Tumor and Anti-Proliferative

Ceramide and DAG, linked by the sphingomyelin (SM) synthase pathway, are two-second messengers that control cell proliferation and growth arrest in opposing ways [87]. In SM and DAG, SMS transports the phosphocholine group from the PC to the ceramide [88]. SMS comes in two varieties: SMS1 is located in the Golgi apparatus, while SMS2 is found in the plasma membrane [89,90]. D609 inhibits both forms of SMS. By preventing ceramide injection into SM, inhibiting SMS elevates ceramide levels. Thanks to SMS inhibition, D609 prevented the proliferation of ßFGF-stimulated astrocyte [16,91]. According to one study, D609 decreased non-neuronal cell-line proliferation without triggering cell death [67]. In BV-2 microglia, D609 therapy elevated the expression of ceramide and p21 levels [50,92]. D609 also hypophosphorylated Rb, causing cell-cycle inhibition in the G0/G1 step, and a decrease in proportion of cells in the S-Phase [50]. Ceramide′s significance in D609-induced cell-cycle arrests is supported by the exogenous C8-ceramide investigations [50].

D609 regulates ceramide formation and cell death caused by death receptors [93,94]. Nontoxic concentrations of D609 inhibit sphingomyelin synthase and glucosylceramide synthase in Jurkat cells [95]. FasL-induced caspase activation and apoptosis were significantly enhanced by D609 [96,97]. Since the authors claim that Bcl-xL overexpression caused D609 outcomes, mitochondrial events were likely involved. The authors conclude that D609 causes cell death in T lymphocyte cells by acting downstream of caspases 8. They believe that in Fasl-induced cell death, a rise occurs due to D609 inhibition of ceramide transfer to complex sphingolipids [98]. Anti-HER2 medicines, which block the tumorigenic actions of HER2, do not significantly affect the treatment of HER2-positive EOC (epithelial ovarian cancer) [99,100]. Preclinical trial models can be utilized to assess the molecular processes causing HER2 overexpression and oncogenicity, giving rise to new EOC treatments. The enhanced HER2 expression in breast cancer cells is regulated by PC-PLC [14]. Researchers found that the inhibition of PC-PLC may be a good target for the tumorigenic effects of increased HER2 expression in EOC41 cells [44].

### 4.5. Neuroprotective

Evidence suggests that oxidative stress has a role in the pathogenesis of Alzheimer′s disease [101]. Amyloid-peptide accumulation causes a cascade of oxidative neuron damage and eventually leads to neuronal death, one of the hallmarks of Alzheimer′s disease [102,103]. Amyloid-peptide is senile plaque’s major component. It causes damage to nucleic acids, proteins, and membrane lipids in neurons by generating free radicals [104,105]. As a result, interest is growing in antioxidant chemicals as they play a protective role in the treatment of AD and other disorders of oxidative stress [106,107]. Amid the various antioxidant medications, “thiol-delivering” compounds have received much attention. PC-PLC is inhibited by D609, a compound that mimics the action of glutathione. Recent studies have shown that it can inhibit phosphatidylcholine-specific phospholipase C [108,109]. Another study assesses the efficiency of D609 to protect synaptosomes in vivo from amyloid-peptide caused by oxidative stress [110,111]. After gerbils were fed D609 or saline solution, synaptosomes were extracted from their brains. The ex vivo treatment of gerbils with synaptosomal preparations derived from D609-injected gerbils with amyloid peptide (1–42) significantly decreased oxidative stress parameters, such as reactive oxygen species, lipid peroxidation (4-hydroxy-2-nonenal), protein oxidation (carbonyl and 3-nitrotyrosine protein), and levels [110]. These findings support the idea that amyloid-peptide-induced free-radical control may be a useful therapeutic tool for treating AD and other related oxidative stress diseases [112]. Based on the above evidence, D609 is a potent antioxidant that may help cure AD and other related oxidative stress diseases [108,110,113,114].

Tricyclodecan-9-yl-Xanthogenate has anti-proliferative, antioxidant, and anti-inflammatory properties. In mitochondria, cellular oxidation of the substrate releases energy in the ATP form [27,115]. This process involves the release of reactive oxygen species (ROS), causing damage to membrane phospholipids, DNA, and proteins, eventually leading to cell death and disease [116,117]. On the other hand, endogenous antioxidants scavenge ROS and prevent cell death [118,119]. D609 was discovered to be a powerful inhibitor of the PCPLC, which is essential for proliferation and cell survival [120,121]. D609 is an effective antioxidant and glutathione mimetic drug in previous studies.

Similarly, there is a substantial amount of literature explaining D609′s anti-proliferative effects in various cells. D609 has been shown in studies to inhibit the proliferation of neural progenitor cells [122,123]. Despite numerous studies, the mechanism of D609-induced cell-cycle arrest remains unclear. Furthermore, only a limited amount of knowledge is available on D609′s antioxidant impact on neural progenitor cells. Researchers could use D609′s neuroprotective and anti-proliferative properties to treat diseases such as stroke and cancer, which both need ATP for survival and proliferation if they could determine how it works on its potential targets. In studies on the function of the ATP content and cytochrome c oxidase of neural progenitor cells, D609′s antioxidant properties were also highlighted. In this research, an in vitro cell culture model of neural progenitor cells obtained from adult rat brains was used [110,124,125]. D609 possesses antioxidant properties, with its neuroprotective and antiproliferative properties encompassing a wide range of cells. D609 was previously found to reduce neural progenitor cell distribution. The antioxidant properties of D609 have been used to assess cell oxidation and calculate cells′ ATP content in the neural progenitor cells extracted from rat’s brains in the subventricular area. D609 reduces the neural progenitor cells’ ATP content by about 40%, which may inhibit cellular metabolic capability [124,126]. COX, also called ETC complex IV, is a terminal enzyme that causes the oxidation of substrates to generate energy for cellular activity [127]. Modulating COX activity can thus interfere with ATP production, influencing cell proliferation. After the incubation of D609 neural progenitor cells, cytochrome C oxidase activity was found to decrease, supporting this hypothesis [128,129]. According to these findings, D609 can inhibit cytochrome C oxidase activity and, as a result, ATP synthesis in neural progenitor cells [129].

### 4.6. Cholinergic Neuron Differentiation

A cholinergic neuron is a nerve cell that sends signals primarily via acetylcholine (ACh). Cholinergic neurons, which primarily use the neurotransmitter acetylcholine (Ach) for message transmission, play a crucial role in memory, locomotion, and behavioral response [130,131]. Cholinergic neuron loss causes a decline in choline acetyltransferase (ChAT) function, which causes motor nerve degeneration and cognitive dysfunction, as seen in AD [132,133]. A new treatment for cholinergic neuron loss is yet to be found, despite the use of cell-based therapies and nerve transplants to treat a variety of neurological disorders [134]. Several stem-cell-based therapies, including motor nerve disorders and Alzheimer′s disease, have recently been proposed as experimental therapies to alleviate the pathophysiology of cholinergic nerve disorders [134,135]. The tricyclodecane-9-yl-xanthogenate (D609) neuronal induction approach was used to successfully differentiate hDPSCs-cryo into cholinergic neurons (DF-chN) [136]. Motor nerve regeneration on an unprecedented scale was observed when DF-chN was inserted in vivo into experimental rats with sciatic nerve defects [136]. The simple inhibitor of phosphatidylcholine-specific C phospholipase, D609, has previously been shown to distinguish bone marrow MSCs (BMSCs) from neuron-like cells [137]. In BMSCs, D609 induces neuron-like cells with cholinergic neuronal properties; however, the mechanism underlying D609 neurogenic induction is unknown [138]. There is another belief that the treatment of D609 in BMSCs blocks PC-PLC activity, while elevated levels of HSP70 trigger the activation of transcription regulator B-cell translocation gene 2 (BTG2), resulting in cholinergic neuronal differentiation based on the number of responsive neuronal-specific genes [139].

D609 therapy has also been shown to reduce mesodermal and endodermal differentiation gene expression while increasing neuroprotection, neuronal differentiation, and cholesterol synthesis gene expression [136]. The formation and maintenance of the myelin sheath require cholesterol. In addition, differentiated cholinergic neuron relocation increases functional neuron regeneration and protection in animals with spinal cord injuries in vivo models. As a result, treating D609 with BMSCs is a quick and easy way to induce a cholinergic neuron induction [138]. According to Soomi Jang et al. (2018), treatment with D609 caused stem cells (derived from cryopreserved dental pulp) to appropriately transform into cholinergic neurons [138]. These cholinergic neurons, differentiated from dental pulp stem cells, had morphological properties similar to those of neurons, such as a neuron body and axonal fiber, as well as positive mRNA and protein expression of cholinergic neuronal markers [136].

Since bone marrow stromal cells (BMSCs) have a low degree of neuronal differentiation in vivo, raising the number of BMSC-derived neurons to treat neurological disorders is critical. According to Chunhui Sun et al., D609, an inhibitor of PC-PLC, stimulated BMSCs to differentiate between neuron-like cells in vitro. The neuronal form, on the other hand, was not exact. It is still uncertain whether these neuron-like cells restore neuronal dysfunction by exhibiting functional neuronal physiological activities. Chunhui Sun et al. assessed their properties by noting the neurotransmitters involved in neuron function and calcium to answer these questions. In vitro, both cells had to function cholinergic neurons, according to the findings. The regeneration of mice with injured spinal cords was aided by the transplantation of such cholinergic neuron-like cells, which were more effective than BMSCs. When cholinergic neuron-like cells derived from BMSC were injected, the proportion of cholinergic neurons increased, indicating a high degree of in vivo differentiation. The cholinergic neuron percentage in host cells rose, acetylcholine synthesis increased, and neurofilament preservation was seen in the lesions of animals injected with BMSC-derived neurons, implying neuronal protection. The results of the study provide a clear method for converting BMSCs into cholinergic neuron-like cells, as well as a potential treatment plan for spinal injuries [138]. In spinal-cord-injured mice, D609 allows BMSCs to differentiate into cholinergic neuron-like cells, and their distribution promotes the functional regeneration and defense of the neurons. This study revealed a new approach for BMSCs to obtain cholinergic neuron-like cells, as well as novel treatment options for neurological disorders. An overview of the pharmacological properties of D609 is presented in Table 1.

## 5. Conclusions

This review focuses on the multiple and diverse pharmacological activities of D609, including its inhibition of cancer proliferation, inflammatory cascade, oxidation stress, and neuroprotective effects. It seems that D609 regulates multiple pathways by targeting SMS/PC-PLC. It promotes the de novo synthesis of ceramide by stimulating SPT and has mimetic antioxidant/glutathione effects due to the presence of the thiol feature. Moreover, D609 attenuates oxidized PC (OxPC) and ROS development via its glutathione/antioxidant mimetic properties, thereby ameliorating CNS pathologies. Recently, it has been shown that D609 defends primary neuronal cultures against amyloid β-peptide-induced neurotoxicity and oxidative stress in vitro/in vivo synaptosomes.

Furthermore, researchers believe that D609 treatment increases HSP70, which stimulates the transcription regulator B-cell translocation gene 2 (BTG2), triggering cholinergic neuronal differentiation by increasing the level of receptive neuronal-specific genes. By suppressing the proliferation of microglia/macrophages, D609 can reduce free radical formation, especially ROS and oxidized PC (OxPC). This also inhibits the replication of herpes simplex virus type 1 without causing cytotoxicity, and has a diminishing effect on nitrite and peroxide expression, while it increases superoxide dismutase and lowers iNOS expressions. D609 also activates NF-kB transcription, blocks the stimulation of PARP-1, and causes microglial morphological transformation, significantly improving the caspase activation and apoptosis triggered by FasL.

Hence, through various pharmacological activities, this compound can be used as a multitarget approach to combat various diseases. Further studies are needed to determine the exact preventive mechanism of action of D609 in various diseases; this review will help academics to conduct more detailed research on this topic.

## Figures and Tables

**Table 1:** Pharmacological properties of D609.

Pharmacological Properties	Target Disease	Model Used	Mechanism of Action	Reference
Antioxidant activity	Alzheimer′s disease (AD)	Isolated gerbil brain mitochondria (50 mg/kg body wt.)	Increased activity of glutathione S-transferase, glutathione peroxidase, and glutathione reductase	[18]
	Oxidative stress/ionizing radiation-induced oxidative damage	In vitro	D609 inhibited the Fenton reaction-induced oxidation of dihydrorhodamine 123. (1) Production of reactive oxygen species; (2) decrease in intracellular reduced glutathione; (3) oxidative damage to proteins and lipids; and (4) activation of nuclear factor-κB.	[17]
	Oxidative stress induced by Ionizing radiations	In vivo/mouse model (50 mg/kg i.v)	Inhibited IR-induced cellular oxidative stress.	[17]
	Aβ(1–42)-induced cytotoxicity/Alzehimer’s disease	Aβ(1–42)-induced oxidative cell toxicity in cultured neurons	D609 significantly attenuated Aβ(1–42)-induced cytotoxicity, intracellular ROS accumulation, protein oxidation, lipid peroxidation, and apoptosis.	[26,108,140]
	Respiratory burst induced by H_2_O_2_	Alveolar macrophage respiratory burst (100 µM D609)	D609 has potential as an antioxidant due to its dithiocarbonate functional group, which allows it to slowly react with H2O2 and rapidly reduce cytochrome *c*, which interferes with a common assay for the respiratory burst.	[141]
	Alzheimer′s disease (AD)	Abeta (1–42) induced modulation in phospholipid asymmetry in the synaptosomal membranes (50 mg/kg body wt.)	Aß induced loss of phospholipid asymmetry	[142]
	Age-related macular degeneration	Sodium Iodide induced AMD mouse model	Increased expression of metallothionein	[19]
	Synaptosomal lipid peroxidation (TBARs), protein oxidation (protein carbonyls), and protein conformation	Synaptosomes	Xanthates scavenge hydroxyl radicals and hydrogen peroxide, form disulfide bonds (dixanthogens), and react with electrophilic products of lipid oxidation (acrolein) in a manner similar to GSH	[143]
Antiviral activity	Respiratory syncytial (RS) virus growth	Human epithelial (Hep 2) cells	Compound affects the relative proportion of viral proteins and the phosphorylation of P protein	[75]
	Herpes diseases	Herpes simplex virus type 1	It inhibits the protein kinases and protein phosphorylation affecting a late step in HSV replication	[76]
	HIV	HIV-infected KE37-1 cell line	Inhibition of HIV-1 Replication	[72]
	Various DNA and RNA virus	Herpes simplex virus types 1 and 2, Bovine papilloma virus	Replication blocked at the DNA and RNA level both early and late after infection. Episomal bovine papilloma virus DNA replication and transcription are also inhibited in transformed cells	[71]
	Vesicular Stomatitis	Vesicular Stomatitis Virus	Inhibition of the phosphorylation of the regulatory non-structural protein	[144]
	Transformation of cells in cancer cells	Simian Virus 40	Inhibition of topoisomerase I by more than 1000-fold was detected	[145]
Anti-inflammatory activity	Inflammatory diseases, such as atherosclerosis and septic shock	LPS-induced inflammation in vascular endothelial cells (10 mg/kg)	PC-PLC inhibition. Inhibited LPS induced IL-8 and MCP-1	[146]
	Inflammation	Lipopolysaccharide (LPS)-stimulated macrophages	LPS-induced ERK kinase activation was inhibited, LPS-induced PIP3 kinase activation reduced cytokine expression	[147,148,149]
	Autoimmune uveitis	Experimental melanin protein-induced uveitis (EMIU)	Inhibits inducible nitric oxide synthase (iNOS) induction, a specific inhibitor of phosphatidylcholine-specific phospholipase C	[80]
	Pulmonary edema	ASM-deficient or wild-type control mice with PAF and assessed for the development of pulmonary edema	Acid sphingomyelinase (ASM)-dependent production of ceramid, activation of the cyclooxygenase pathway	[150]
Anti-tumour and anti-proliferative activity	Glial cell proliferation	Murine BV-2 microglia cell line	Ceramide and cell-cycle inhibition, inhibiting SMS can increase ceramide levels, which can inhibit cell proliferation.	[50]
	Metastatic breast cancer cells	Human MDA-MB-231 cells	Inhibition of phosphatidylcholine-specific phospholipase C, downregulates HER2 overexpression on plasma membrane of breast cancer cells	[7,14]
	Human epithelial ovarian cancer cells	OVCAR3 and SKOV3 cancer cells	PC-PLC inhibition	[151,152]
	Fibrosarcoma	Mouse fibrosarcoma cells L929, Wehi164	Through PC-PLC, Anti-inflammatory action, TNF antagonist	[44]
	Leukemia	U937 human monocytic leukemia cells	Sphingomyelin synthase inhibition	[43]
	Cellular proliferation of neural progenitor cells	Neural progenitor cells	Decreasing the ERK-mediated expression of cyclin D1, Decreased the phosphorylation of extracellular signal-regulated kinase (ERK) but not Akt	[153]
Neuroprotective activity	Age-related macular degeneration Retinal pigmented epithelium (RPE) cell death	SI-induced AMD mouse model	Attenuated excessive reactive oxygen species (ROS) and prevented severe mitochondrial loss, increased the expression of metallothionein.	[19]
	Alzheimer’s disease (AD)	AβPP/PS1 transgenic mouse model	Reduced β-secretase 1 level and decreased amyloidogenic processing of AβPP, consequently reducing Aβ deposition in the mice. Reduced oxidative stress.	[70]
	Brain injury	Wistar rats, cultures Obtained from striatum and hippocampus	Inhibitor of phosphoinositide phospholipase C suggesting that TNFαsignaling in neurons involved the acidic sphingomyelinase	[154]
	Neurodegenerative disorders	Gerbil brain mitochondria	Increased activity of glutathione S-transferase, glutathione peroxidase, and glutathione reductase in brain	[18]
	Stroke	Transient middle-cerebral-artery occlusion (tMCAO)	D609 provides benefits after stroke through inhibition of SMS, increased ceramide levels, and induction of cell-cycle arrest by up-regulating p21 and causing hypophosphorylation of Rb	[16]
	Neuroprotective effect	neural progenitor cells isolated from the subventricular zone of the rat brain	D609 could inhibit the activity of cytochrome C oxidase and subsequent ATP synthesis in neural progenitor cells.	[129]
Cholinergic-neuron differentiation activity	Differentiation of cells	Cell differentiation in vascular endothelial cells (VECs) and marrow stromal cells (MSCs).	D609 induces VECs and MSCs differentiation into neuron-like cells.	[155]
	Protocols for cholinergic neuron differentiation	Dental pulp derived MSCs (DPSCs) were used, DPSCs were cultured in serum-free ADMEM containing 15 µg/mL of D609 (tricyclodecan-9-yl-xanthogenate) for 4 days	Neuron-like morphologies with upregulated cholinergic neuron-specific markers, such as ChAT, HB9, ISL1, BETA-3, and MAP2, both at mRNA and protein levels, were observed	[137]
	Bone marrow stromal cells (BMSCs) differentiate into neuron-like cells	Bone-marrow stromal cells (BMSCs)	Inhibition of phosphatidylcholine-specific phospholipase C (PC-PLC) by D609 leads to BMSCs’ differentiation into cholinergic neuron-like cells, Hsp70 participated in the neural differentiation of BMSCs directly through Btg2.	[139]

## Data Availability

Not applicable.
